# Supplementary material for: Aerobiological Monitoring and Metabarcoding of Grass Pollen
Source: Plants (Basel). 2023 Jun 17;12(12):2351. doi: 10.3390/plants12122351 (PMC10302275; doi:10.3390/plants12122351)
Supplement: Supplementary file 1 [file plants-12-02351-s001.zip › plants-2393737-supplementary material.pdf]

Supplementary Materials

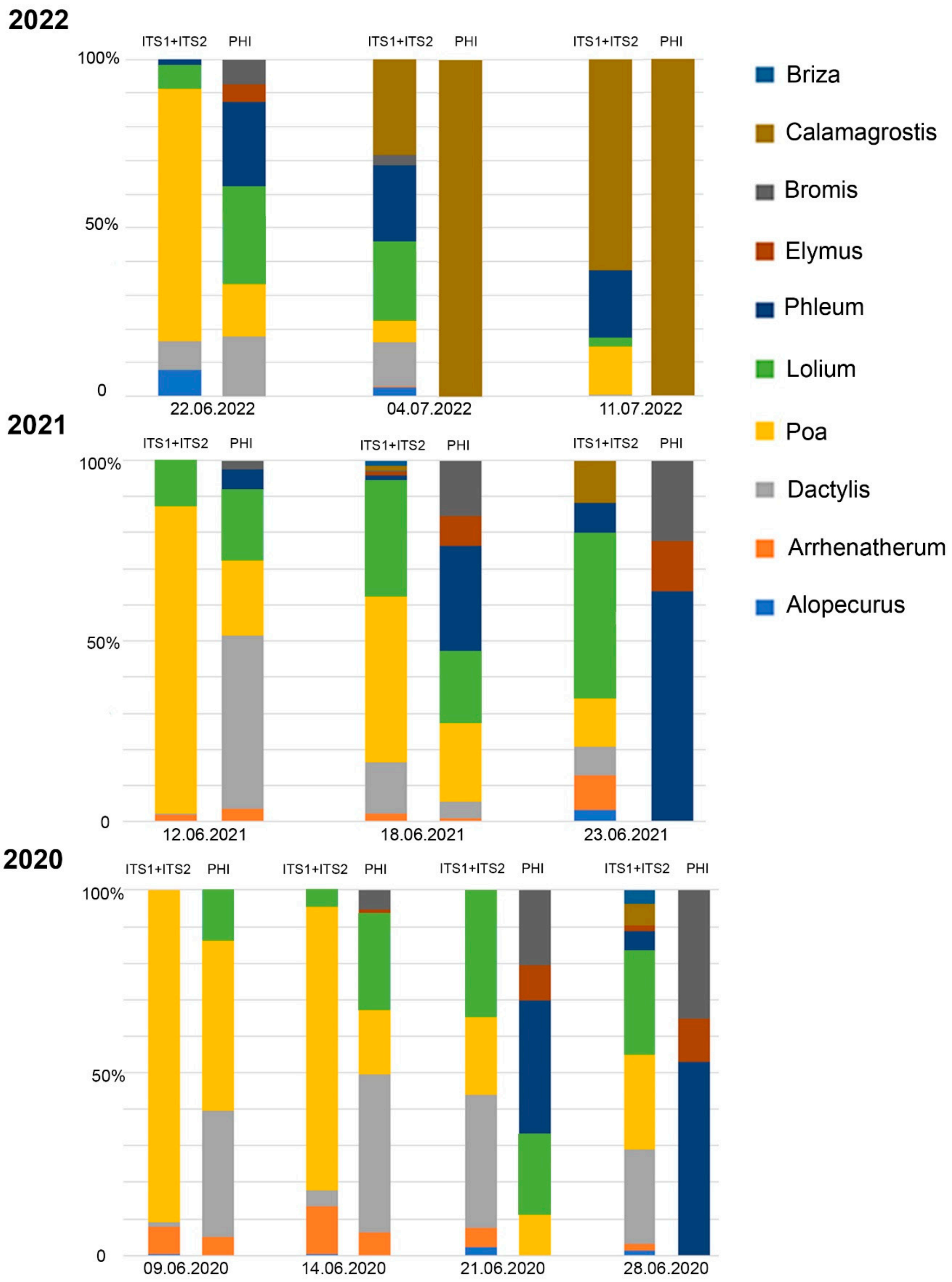

Figure S1. Comparison of metabarcoding results and phenological observations, Moscow 2020-2022. PHI – phenological index

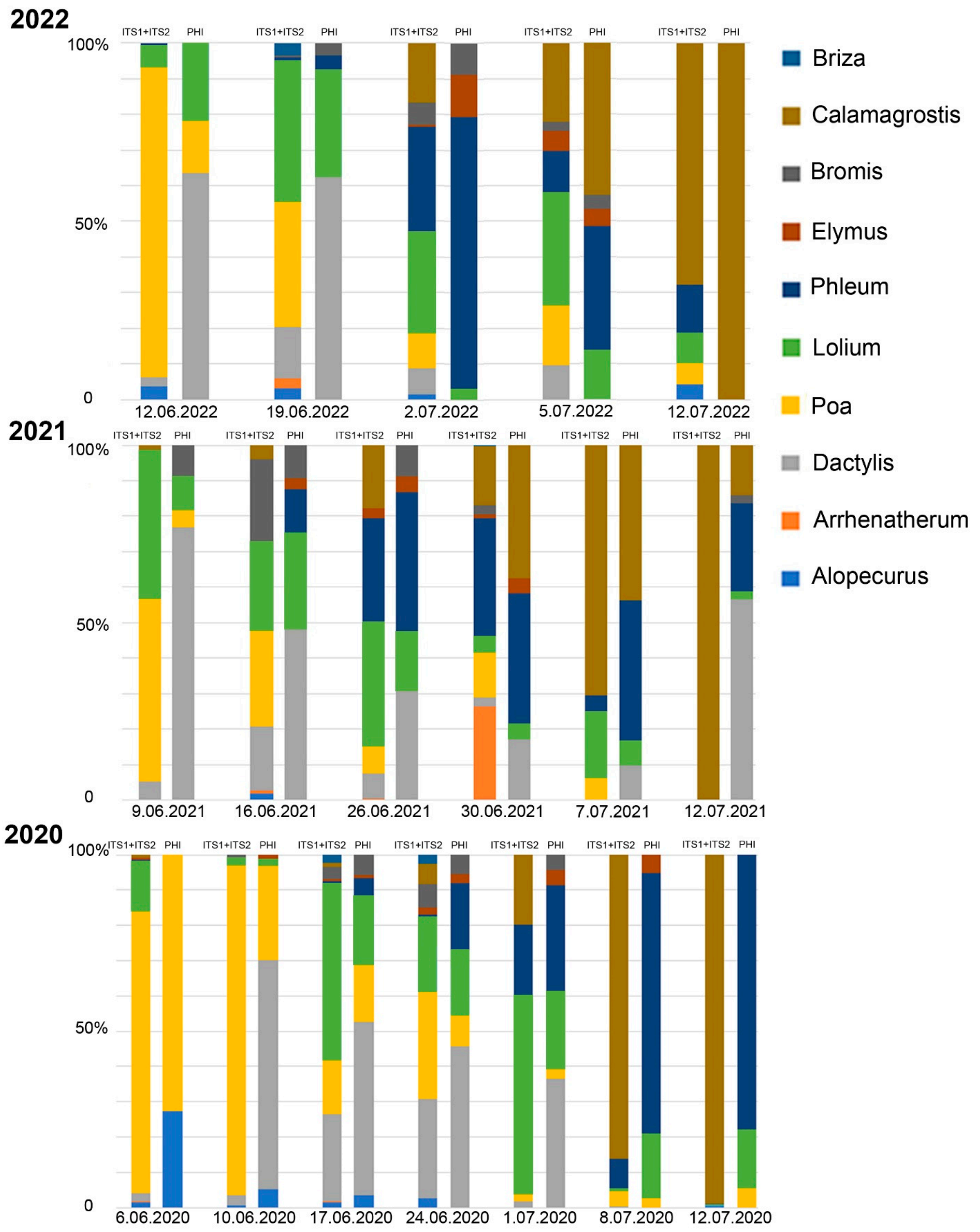

Figure S2. Comparison of metabarcoding results and phenological observations, Ryazan, 2020-2022. PHI – phenological index

Table S1. List of samples.

Moscow			Ryazan		
2020	2021	2022	2020	2021	2022
p1 09-10.06	p23 12-13.06	p35 21-22.06	p48_06-07.06	p85_07-08.06	p113 12-13.06
p2 10-11.06	p24 13-14.06	p36 22-23.06	p49_07-08-06	p86_08-09.06	p114 13-14.06
p3 11-12.06	p25 14-15.06	p37 23-24.06	p50_08-09.06	p87_09-10.06	p115 14-15.06
p4 12-13.06	p26 15-16.06	p38 24-25.06	p51_09-10.06	p88_10-11.06	p116 15-16.06
p5 13-14.06	p27 16-17.06	p39 28-29.06	p52_10-11.06	p89_11-12.06	p117 16-17.06
p6 14-15.06	p28 17-18.06	p40 29-30.06	p53_11-12.06	p90_12-13.06	p118 17-18.06
p7 15-16.06	p29 18-19.06	p41 30.06-1.07	p54_12-13.06	p91 13-14.06	p119 18-19.06
p8 16-17.06	p30 19-20.06	p42_01-02.07	p55 13-14.06	p92 14-15.06	p120 19-20.06
p9 17-18.06	p31 20-21.06	p43_02-03.07	p56 14-15.06	p93 15-16.06	p121 02-03.07
p10 18-19.06	p32 21-22.06	p44_03-04.07	p57 15-16.06	p94 16-17.06	p122 03-04.07
p11 19-20.06	p33 22-23.06	p45_04-05.07	p58 16-17.06	p95 17-18.06	p123 04-05.07
p12 20-21.06	p34 23-24.06	p46_10-11.07	p59 17-18.06	p96 18-19.06	p124 05-06.07
p13 21-22.06		p47_11-12.07	p60 18-19.06	p97 19-20.06	p125 06-07.07
p14 22-23.06			p61 19-20.06	p98 26-27.06	p126 07-08.07
p15 23-24.06			p62 20-21.06	p99 27-28.06	p127 08-09.07
p16 24-25.06			p63 21-22.06	p100 28-29.06	p128 09-10.07
p17 25-26.06			p64 22-23.06	p101 29-30.06	p129 10-11.07
p18 26-27.06			p65 23-24.06	p102 30.06-1.07	p130 11-12.07
p19 27-28.06			p66 24-25.06	p103_01-02.07	p131 12-13.07
p20 28-29.06			p67 25-26.06	p104_02-03.07	
p21 29-30.06			p68 26-27.06	p105_03-04.07	
p22 30.06-1.07			p69 27-28.06	p106_04-05.07	
			p70 28-29.06	p107_07-08.07	
			p71 29-30.06	p108_08-09.07	
			p72 30.06-1.07	p109_09-10.07	
			p73_01-02.07	p110_10-11.07	
			p74_02-03.07	p111_11-12.07	

			p75_03-04.07	p112_12-13.07	
			p76_04-05.07		
			p77_05-06.07		
			p78_06-07.07		
			p79_07-08.07		
			p80_08-09.07		
			p81_09-10.07		
			p82_10-11.07		
			p83_11-12.07		
			p84_12-03.07		

Table S2. List of species observed on sample plots. All species names are according to https://powo.science.kew.org/ Dominated species are in **bold.**

	Moscow									Ryazan′				
	№ of sample plot									№ of sample plot				
Species	1	2	3	4	5	6	7	8	9	1	2	3	4	5
Alopecurus pratensis L.	x									x				
Arrhenatherum elatius (L.) P. Beauv. ex J. & C. Presl.	x			**x**		**x**								
Briza media L.	x													
Bromus inermis Leyss.	**x**		x							**x**			x	x
Calamagrostis epigejos (L.) Roth	x	x	**x**				x						x	x
Dactylis glomerata L.	x	**x**	x	x	**x**	x	x		**x**	x	**x**	**x**	x	x
Elymus repens (L.) Gould	x	**x**	x	x	x		x			x	x			x
Lolium pratense (Huds.)Darbysh.	**x**	x	**x**	x	x	x	x	x	x	x	**x**	x	x	x
Lolium perenne L.			x	x				x	x	x		x		
Phleum pratense L.	x	x	**x**				x	**x**	x	x	x	x	x	x
Poa annua L.						x						x	x	x
Poa pratensis L.	x	**x**		x	**x**	x	**x**	x	x	**x**	**x**		x	x
Poa trivialis L.	x	**x**	x				x					x		
